# Triazole resistance mediated by mutations of a conserved active site tyrosine in fungal lanosterol 14α-demethylase

**DOI:** 10.1038/srep26213

**Published:** 2016-05-18

**Authors:** Alia A. Sagatova, Mikhail V. Keniya, Rajni K. Wilson, Manya Sabherwal, Joel D. A. Tyndall, Brian C. Monk

**Affiliations:** 1 Sir John Walsh Research Institute, University of Otago, Dunedin, New Zealand; 2 New Zealand’s National School of Pharmacy, University of Otago, Dunedin, New Zealand; 3 Department of Oral Sciences, University of Otago, Dunedin, New Zealand

## Abstract

Emergence of fungal strains showing resistance to triazole drugs can make treatment of fungal disease problematic. Triazole resistance can arise due to single mutations in the drug target lanosterol 14α-demethylase (Erg11p/CYP51). We have determined how commonly occurring single site mutations in pathogenic fungi affect triazole binding using *Saccharomyces cerevisiae* Erg11p (ScErg11p) as a target surrogate. The mutations Y140F/H were introduced into full-length hexahistidine-tagged ScErg11p. Phenotypes and high-resolution X-ray crystal structures were determined for the mutant enzymes complexed with short-tailed (fluconazole and voriconazole) or long-tailed (itraconazole and posaconazole) triazoles and wild type enzyme complexed with voriconazole. The mutations disrupted a water-mediated hydrogen bond network involved in binding of short-tailed triazoles, which contain a tertiary hydroxyl not present in long-tailed triazoles. This appears to be the mechanism by which resistance to these short chain azoles occurs. Understanding how these mutations affect drug affinity will aid the design of azoles that overcome resistance.

Fungal infections are estimated to directly affect approximately one billion people globally and also have the potential to affect food security, especially in temperate and tropical climates. The increased incidence of potentially lethal invasive fungal infections (IFIs) and the emergence of resistant fungal pathogens are growing concerns^1^,^2^.

Fungal infections of humans that require medical intervention affect the immune-deficient, such as the very young (premature infants), females of reproductive age, the elderly and the debilitated. The most susceptible individuals have co-morbidities such as AIDS and cancer, or are patients who are medically immune suppressed. Members of the fungal species *Candida*, *Aspergillus* and *Cryptococcus* are the most prominent causes of IFIs in humans^3^,^4^. For example, in Sub-Saharan Africa where AIDS is endemic, infection by *Cryptococcus neoformans* is estimated to cause over 500,000 deaths each year^5^. *Candida albicans* is the 4^th^ leading cause of blood stream infections detected in the United States^6^ while *Aspergillus fumigatus* infections have a high mortality rate among transplant patients^7^.

Fungal infection of plants, crops, fruit and stored produce is a major problem, especially in temperate and tropical climates. This problem is likely compounded by extensive monoculture, where natural barriers to fungal infection are insufficient and the widespread application of fungicides is required^8^ e.g. for control of the soybean phytopathogen *Phakopsora pakyrhizi* or the wheat pathogen *Zymoseptoria tritici* (previously known as *Mycosphaerella graminicola*). Multiple generations of phytopathogen-targeting triazole agrochemicals have been used since the 1970s to counter shifts in antifungal susceptibility elicited by exposure to earlier azoles.

The triazole drugs, the largest class of antifungals used in the clinic or as agricultural fungicides, are becoming less effective in treating mycoses of humans and animals and in preventing the devastating effects of phytopathogens on crop production. This has occurred because of the emergence of less susceptible fungal strains^9^. Resistance to azoles used in medicine has arisen, in part, because of the use of prophylaxis with susceptible patients and prolonged treatment courses^10^,^11^. A contribution to this problem by azole-based agrochemicals was recognised recently, with these fungicides shown to induce cross-resistance to medical triazoles in *A. fumigatus* and *Candida glabrata* strains^12^,^13^. There is an increasingly urgent need to develop antifungals and agrochemicals capable of overcoming triazole resistance.

A common mechanism of resistance among fungal pathogens occurs due to mutations in the enzymatic target of azole drugs, the cytochrome P450 lanosterol 14α-demethylase (denoted as Erg11p or CYP51). Azoles inhibit the demethylase by coordinating to the heme iron in the active site via a heterocyclic nitrogen atom of an imidazole, triazole or tetrazole ring^14^,^15^,^16^. In *C. albicans* Erg11p (CaErg11p) the Y132F/H mutations have frequently been detected in clinical isolates and lead to a 4-fold increase in resistance to fluconazole (FLC)^17^,^18^. Homologous mutations occur in the CYP51 genes of other fungal pathogens of man and plants (Fig. 1a)^8^,^19^,^20^. For example, the Y145F mutation in *C. neoformans* CYP51^21^ and Y136F mutation in *Histoplasma capsulatum* CYP51^22^ both cause resistance to the short-tailed triazoles FLC and voriconazole (VCZ) but not to the long-tailed triazoles posaconazole (PCZ) or itraconazole (ITC, Fig. 1b). Similarly, the mutations Y137F in *Z. tritici* CYP51^23^ and Y136F in *Uncinula necator* CYP51^24^ confer reduced susceptibility to the short-tailed azole fungicide triadimenol. In *A. fumigatus* the homologous CYP51A Y121F mutation can occur alone^25^ or together with the T289A mutation and tandem repeat 46 (TR_46_) in the promoter region^26^. The Y121F mutation confers resistance to VCZ but not ITC or PCZ^25^ while the TR_46_ Y121F T289A mutation is associated with failure of voriconazole therapy and a slightly reduced susceptibility to ITC and PCZ^26^.

The selection pressure exerted by individual triazole drugs can differ. For example, the *Z. tritici* CYP51 Y137F mutation arose after the introduction of triadimenol but essentially disappeared in the field following the introduction of prothioconazole^27^, a pro-drug that is metabolised to the active short-tailed triazole agrochemical prothioconazole-desthio^28^. The accumulation of multiple mutations in CYP51 can lead to significant reductions in triazole susceptibility. For example the Y131F I475T combination of mutations has been detected in CYP51 of the strains of the phytopathogen *P. pachyrhizi*^29^. The comparable double mutation Y132H I471T is found in Erg11p of the triazole resistant Darlington strain of *C. albicans*^30^.

Until recently, a paucity of structural information on fungal lanosterol 14α-demethylase has meant that the molecular mechanisms determining the reduced susceptibility conferred by mutations in fungal lanosterol 14α-demethylase were not well understood. We have reported high-resolution structures of full-length *Saccharomyces cerevisiae* hexahistidine-tagged Erg11p (ScErg11p6 × His) in complex with its substrate lanosterol (1.9 Å, PDB ID: 4XLJ) and the long chain azole itraconazole (2.1 Å, PDB ID: 5EQB)^31^. We have also described a high-resolution (2.05 Å) structure of ScErg11p6 × His in complex with FLC (PDB ID: 4WMZ)^32^. Based on the latter structure, the ScErg11p Y140F/H mutations were proposed to potentially modify a water-mediated hydrogen bond network involving the tertiary hydroxyl group of FLC, thus weakening the binding of the drug. In the present study, new high-resolution X-ray crystal structures of the ScErg11p6 × His Y140F mutant in complex with ITC, FLC, VCZ and PCZ and the Y140H mutant in complex with FLC and ITC, along with the wild type ScErg11p6 × His in complex with VCZ, provide evidence that disruption of this hydrogen bond network leads to weaker drug binding.

## Results

### Quantitation of the expression of ScErg11p6 × His in crude membrane fractions

The strains used in the present study are shown in Supplementary Information, Supplementary Table S1.

Coomassie blue R250 stained SDS-polyacrylamide gel profiles (Supplementary Fig. S1a) and western blot analysis (Supplementary Fig. S1b) of crude membrane fractions obtained from yeast overexpressing ScErg11p6 × His, ScErg11p6 × His Y140F or ScErg11p6 × His Y140H show that the mutant enzymes were expressed in this fraction at levels at or near wild type enzyme. Analysis of the Ni-NTA and affinity-purified 61 kDa band by tryptic digestion and mass spectrometry showed at least 78% coverage of the primary sequence of ScErg11p6 × His and confirmed the presence of the expected mutations at Y140 (Supplementary Figs S2 and S3).

### Azole susceptibilities of strains overexpressing ScErg11p6 × His Y140F/H

Susceptibilities to triazole drugs of *S. cerevisiae* AD3Δ strains overexpressing Erg11p6 × His Y140F/H, but with the native *ERG11* deleted, were measured as liquid MIC_80_ values (Supplementary Table S2). Overexpression of ScErg11p6 × His Y140F in the AD3ΔScErg11p_Y140F strain conferred 2-fold lower susceptibility to FLC than the strain overexpressing wild type ScErg11p6 × His i.e. MIC_80_ of 4.0 μg/ml and 2.1 μg/ml, respectively. The same mutant conferred a 1.7-fold reduction in susceptibility to VCZ. The MIC_80_ values were 0.42 μg/ml for the strain overexpressing ScErg11p6 × His Y140F and 0.25 μg/ml for the strain overexpressing wild type ScErg11p6 × His. The susceptibilities to ITC were comparable for the strains overexpressing the ScErg11p6 × His Y140F, ScErg11p6 × His Y140H or wild type enzyme i.e. 0.09 μg/ml, 0.77 μg/ml and 0.105 μg/ml, respectively. Strain AD3ΔScErg11p_Y140H showed slightly greater susceptibilities to FLC (3.4 μg/ml) and VCZ (0.29 μg/ml) than the Y140F mutant. While strain AD3ΔScErg11p_Y140H showed a reduced susceptibility to FLC, its susceptibility to VCZ was comparable to the control strain overexpressing the wild type enzyme.

### Spectral characterisation of triazole binding to wild type and mutant ScErg11p6 × His

**Figure 2:**
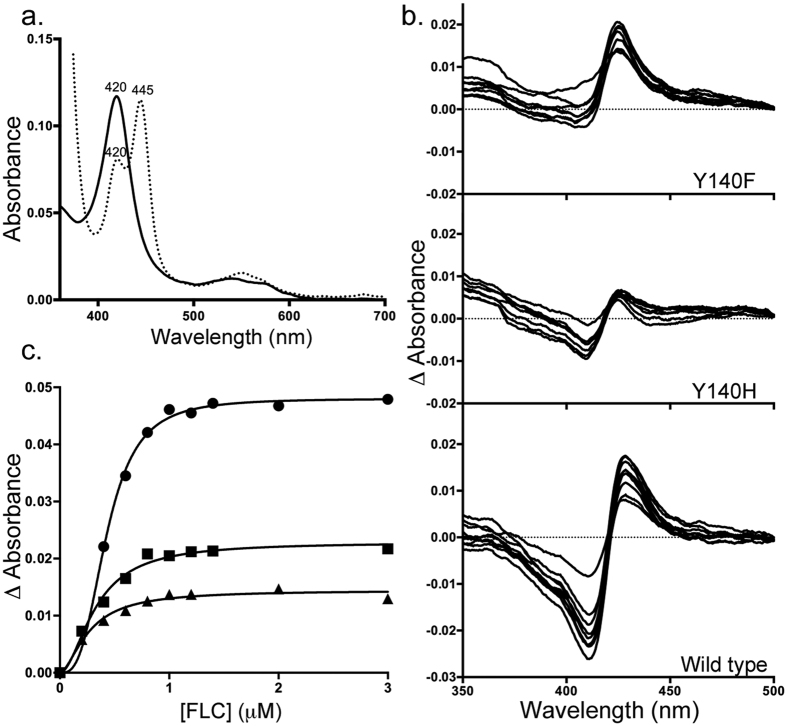
Spectral characterisation of ScErg11p6 × His Y140F/H mutants. (**a**) The absolute (continuous line) and CO bound (dotted line) spectra of ScErg11p6 × His Y140F mutant. (**b**) Type II difference spectra for FLC binding to ScErg11p6 × His wild type and mutant enzymes. (**c**) Fluconazole binding to wild type ScErg11p6 × His (●) ScErg11p6 × His Y140F (■) and ScErg11p6 × His Y140H (▲). All curves best fit the Hill equation.

The absolute absorbance spectra for wild type ScErg11p6 × His showed an oxidised Soret peak at ~417 nm^32^ and for the Y140F/H enzymes the peak is seen at 420 nm (Fig. 2a). Carbon monoxide difference spectra^33^ obtained with dithionite-reduced wild type and mutant enzymes gave a peak at 445 nm which showed that the bulk of each affinity-purified enzyme was functional (Fig. 2a). The presence of a slightly larger shoulder at 420 nm for the mutant enzymes compared to the wild type enzyme^32^ indicated that the affinity-purified mutant enzyme may be less stable than the wild type enzyme.

Ni-NTA affinity purified mutant and wild type ScErg11p6 × His preparations (1 μM) were used to detect and quantitate type II azole binding. The spectral shift of the Soret peak from 417 nm to 421–424 nm in cytochrome P450s is also detected by the type II difference spectra. This change is associated with the replacement of a water molecule coordinated to the heme iron with the azole heterocycle. Type II difference spectra were obtained for FLC, VCZ, ITC and PCZ binding to both mutant enzymes. The binding of FLC is shown as an example (Fig. 2b). The type II difference spectra for the mutant enzymes were less intense than for the wild type enzyme. They gave 2–3-fold smaller differences in absorbance (ΔA_max_), with peaks shifted from 428 nm for wild type enzyme to 424–425 nm for the mutant enzymes plus less well-defined troughs between 406–410 nm (Supplementary Table S3). The type II binding curves obtained using the wild type ScErg11p6 × His and the Y140F/H mutants (1 μM) showed a sigmoidal dose-response (Fig. 2c) that gave best fit using the Hill equation. The resultant [Azole]_0.5_ and *K*_d_ values are listed in Table 1. These values indicate tight binding for each drug tested. The *K*_*d*_ values for the wild type and mutant enzymes were comparable i.e. within the margin of the standard error.

### X-ray crystal structures of ScErg11p6 × His Y140F/H mutants in complex with ITC and PCZ

**Figure 3:**
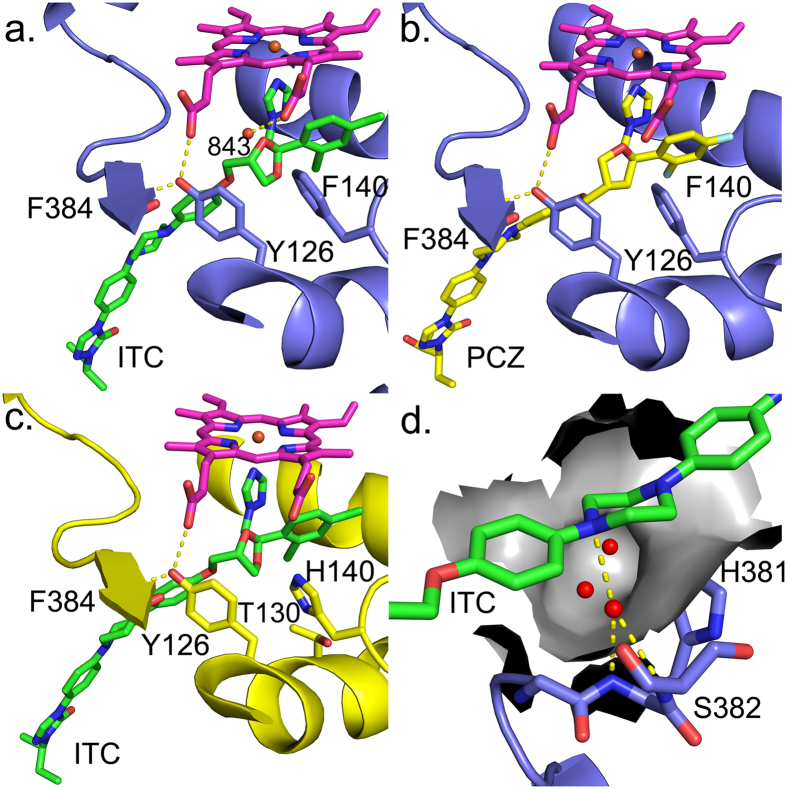
Binding of long-tailed azoles ITC and PCZ to ScErg11p6 × His Y140F/H. (**a**) ScErg11p6 × His Y140F in complex with ITC (PDB ID: 4ZDY) and (**b**) PCZ (PDB ID: 4ZE1) and (**c**) ScErg11p6 × His Y140H in complex with ITC (PDB ID: 4ZE2). Panel (**d**) depicts the hydrophilic pocket (surface representation) in the substrate channel of ScErg11p6 × His Y140F. The hydrogen bonding interactions are shown between the water molecule (930), the N1 of the piperazine ring and the main chain amide groups of H381 and S382. The ScErg11p6 × His Y140F is shown in lilac cartoon and ScErg11p6 × His Y140H is depicted in yellow cartoon. Hydrogen bonds are shown as yellow dashed lines and water molecules as red spheres. ITC (green carbons), PCZ (yellow carbons), the side chains of residues 140 and 126, the backbone of F384, residues H381 and S382 and the heme (magenta) are shown as sticks.

**Figure 4:**
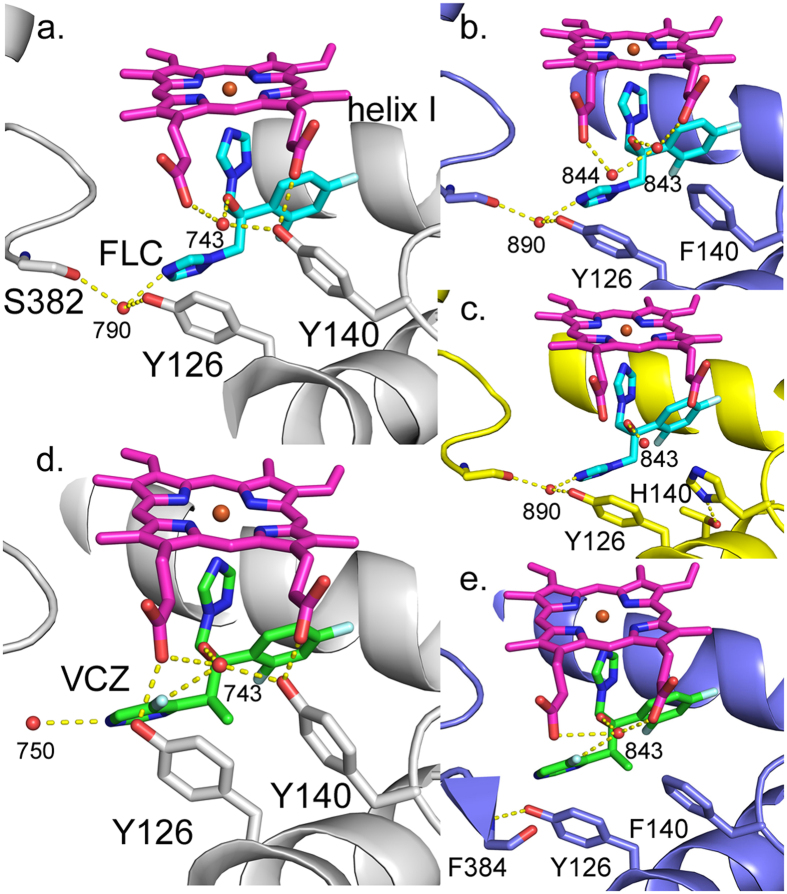
Binding of short-tailed azoles FLC and VCZ to ScErg11p6 × His. Water-mediated interactions are shown for binding of (**a**) FLC and (**d**) VCZ to wild type ScErg11p6 × His (PDB IDs: 4WMZ and 5HS1, respectively). The modified interactions due to the Y140F/H mutations are depicted in panels (**b**) and (**c**) for FLC binding (PDB IDs: 4ZDZ and 4ZE3) and (**e**) for VCZ binding (PDB ID: 4ZE0). The wild type protein is depicted in grey cartoon (PDB IDs: 4WMZ and 5HS1), the Y140F mutant in lilac and the Y140H in yellow. The hydrogen bonds are shown with dashed lines and water molecules as red spheres. FLC (cyan carbons), VCZ (green carbons), the side chains of residue 140, 126, the backbone of S382 and F384 and the heme (magenta) are shown as sticks.

X-ray crystal structures were obtained for ScErg11p6 × His Y140F in complex with ITC and PCZ (PBD IDs: 4ZDY and 4ZE1) at resolutions of 2.02 Å and 2.05 Å (Supplementary Table S4), respectively, and for ScErg11p6 × His Y140H in complex with ITC (PDB ID: 4ZE2) at a resolution of 2.30 Å (Supplementary Table S5). Both the ScErg11p6 × His Y140F and Y140H structures showed the binding of ITC was essentially identical to that seen previously with wild type ScErg11p6 × His (PDB ID: 5EQB, Fig. 3)^31^. In addition to detecting the mutated residue, a water molecule (843) not present in the wild type structure was found between an oxygen of the ITC 1,3-dioxolane group, the propionates of heme rings A and D and the aromatic side chain of the F140 (Fig. 3a) at distances of 4.0, 3.1, 2.5 and 3.3 Å, respectively. Water 843 replaced the phenolic hydroxyl group of Y140 but, because it makes no polar contacts with the inhibitor, it is unlikely to have any effect on ITC binding. The equivalent water molecule was not detected in the structure of ScErg11p6 × His Y140F in complex with PCZ (Fig. 3b) or in ScErg11p6 × His Y140H in complex with ITC (Fig. 3c). Each mutant crystal structure showed the long tail of the triazole ligand bound within the substrate entry channel in an extended conformation, with the piperazine ring in the chair conformation. This conformation was stabilized via a water-mediated hydrogen bond network between the tetrahedral nitrogen atom N1 of the piperazine ring and the main chain amide nitrogens of H381 and S382 (Fig. 3d). The water mediating this hydrogen bond network resides in a hydrophilic pocket that contains two other waters when ITC or PCZ is bound. The binding of the long-tailed triazoles ITC and PCZ to wild type ScErg11p6 × His and the Y140F/H mutants pushes Y126 to a position so it forms hydrogen bonds with the heme ring D propionate group and the amide nitrogen of F384 (Fig. 3). The overall configuration of this residue was found to be less constrained in the presence of FLC, which occupies less space between the two groups (Fig. 4a).

### X-ray crystal structures of ScErg11p6 × His wild type and Y140F/H mutants in complex with FLC and VCZ

The structures of wild type ScErg11p6 × His and the Y140F mutant enzyme in complex with FLC (PDB IDs: 4WMZ and 4ZDZ, respectively) showed the drug to be bound in a similar conformation, with all differences in the interactions between the drug and the enzyme ascribed to the mutation. As described in our previous report^32^, the structure of wild type ScErg11p6 × His in complex with FLC included a water-mediated (743) hydrogen bond network that involves the hydroxyl group of FLC, the hydroxyl of Y140 and the heme ring D propionate (Fig. 4a). In addition, the non-coordinated triazole of FLC and the main chain carbonyl of S382 formed a hydrogen bond network mediated by water 790. This water was not detected in mutant ScErg11p6 × His in complex with ITC or PCZ because the tails of these ligands occupy that space. A water molecule equivalent to water 743 (numbered 843 in the mutant structures) was found in ScErg11p6 × His Y140F in complex with FLC but the hydrogen bond with Y140 was abolished as a result of the Y140F mutation (Fig. 4b, PDB: 4ZDZ). In addition, the Y140F mutation prevented formation of a hydrogen bond with the ring D propionate group of the heme. A new hydrogen bond between the propionate and water 843 now exists. Disruption of the hydrogen bond network by the Y140F mutation results in an additional water molecule (numbered 844), hydrogen bonded to water 843 and heme ring D propionate (Fig. 4b).

Wild type ScErg11p6 × His and ScErg11p6 × His Y140H complexes with FLC show the azole in similar conformations (Fig. 4a,c, PDB IDs: 4WMZ and 4ZE3). Water molecule 843 is present and forms a single short hydrogen bond with the tertiary hydroxyl group of FLC. This water displays a higher B-factor than water 743 in the wild type structure and water 843 in the Y140F mutant structure. A new polar contact (bond distance 2.7 Å) observed between N2 of the imidazole of H140 and the hydroxyl of T130 orients the H140 side chain closer to T130 than is seen with Y140 or F140 side chains (Fig. 4c). For ScErg11p6 × His Y140H in complex with ITC, the orientation of the imidazole ring was similar but the N2 of the H140 imidazole and the hydroxyl of T130 are separated by 3.4 Å.

The crystal structure of wild type ScErg11p6 × His in complex with VCZ (PDB: 5HS1) reveals the azole drug binding in a similar fashion to FLC but with distinct differences due to its structure (Fig. 4d). The pyrimidine ring of VCZ occupies a similar position to the non-coordinating triazole ring of FLC but projects further into the substrate channel. The 5-fluoro substituent of the pyrimidine ring projects towards Y126 forcing this residue close enough to interact with the heme ring A propionate (2.8 Å; as seen in complexes with the long-tailed azoles PCZ and ITC). In the FLC structure (PDB ID: 4WMZ) those two groups are 4.3 Å apart. The key water molecule (743) is present and forms a similar hydrogen-bonding network with the phenolic hydroxyl of Y140, the heme ring D propionate and the tertiary hydroxyl group of VCZ (Fig. 4d). An additional hydrogen bond is present between the 5-fluoro substituent and the key water 743 (3.0 Å), which is shifted slightly towards Y140. The water molecule corresponding to water 790 in the FLC structure is not present at this position due to the projection of the pyrimidine ring. However, a water molecule (750, Fig. 4d) seen in complexes with the long-tailed azoles is also seen here. It forms hydrogen bonds between the pyrimidine nitrogen (N1), the main chain nitrogens of H381 and S382 plus the carbonyl of residue M509 which projects into the binding site cavity (Supplementary Fig. S5). Such flexibility has not been observed in this region previously. In the structure of ScErg11p6 × His in complex with lanosterol the carbonyl of M509 is within 2.9 Å of the tail of the substrate (PDB ID: 4XLJ) while the equivalent residue (M487) in human CYP51 has been implicated in substrate recognition due to its close proximity to the ligand ketoconazole (PDB ID: 3LD6)^34^.

The crystal structure of the ScErg11p6 × His Y140F mutant in complex with VCZ (Fig. 4e, PDB ID: 4ZE0) shows the ligand binds in a similar orientation but slightly deeper into the active site than in the wild type enzyme, with the quaternary carbon shifted ~0.5 Å. The mutation to phenylalanine with the corresponding removal of the hydroxyl group lets water 843 occupy the vacated space and thus allow the ligand to bind deeper. In addition, the plane of the phenyl ring of F140 is slightly shifted away from the ligand compared with the wild type complex with VCZ. Y126 is also in a similar position to that seen in the wild type VCZ complex. The distance between the heme ring A propionate and Y126 is 4.4 Å and the additional water (750) is not seen in the mutant complex. M509 no longer projects into the active site but is adjacent to F236. In comparison to the recent structure of *A. fumigatus* CYP51B in complex with VCZ^35^, the pyrimidine ring is rotated 180°, allowing the ring to approach Y122 (*A. fumigatus* numbering; Y126 in ScERG11p6 × His), while the 5-fluoro substituent is oriented towards F504 in a space in the ScErg11p6 × His structure adjacent to V510.

## Discussion

We have created and analysed the structure and function of surrogate triazole resistance phenotypes in the yeast *S. cerevisiae* by creating Y140F/H single site mutations in lanosterol 14α-demethylase that have homologues in numerous species of pathogenic fungi^9^,^19^. This study shows how alterations to a water-mediated hydrogen-bonding network, between the drug and target protein, can affect the affinity of the short-tailed triazole drugs FLC and VCZ but not the long-tailed triazoles ITC and PCZ. Whole cell assays (MIC_80_ measurements) were used to confirm the reduced susceptibility of ScErg11p6 × His Y140F/H mutants to FLC and VCZ. The <2-fold differences in MIC_80_ values detected using overexpression of wild type and mutant ScErg11p6 × His in the yeast system were not as dramatic as previously reported for the *C. albicans* Erg11p Y132H mutation^17^ but show a similar trend.

Triazole binding studies using affinity-purified wild type and ScErg11p6 × His Y140F/H enzymes gave differences in both absolute absorption spectra prior to the addition of ligand and the magnitude of the change obtained for type II difference spectra after ligand addition. The differences in the oxidised heme Soret peak between the Y140F/H mutant enzymes (420 nm) and the wild type enzyme (417 nm) indicate that the mutations alter the electronic environment of the heme. Disruption of the short hydrogen bond between Y140 and the heme ring D propionate is the common feature that may explain this effect in both the Y140F and Y140H mutants.

The [Azole]_0.5_ and *K*_d_ values obtained from plotting the change in absorbance against the azole concentration with the wild type and mutant enzymes for each triazole drug indicated comparable high affinity binding (Table 1). The apparent lack of correlation between resistant phenotypes and azole affinity for free CYP51 enzyme was previously reported for the *C. albicans* CYP51 Y132H mutant^36^ and the I471T mutant^37^. This may reflect difficulty in extracting the information needed to calculate inhibitor affinities due to the formation of near stoichiometric enzyme-inhibitor complexes. In addition, Warrilow *et al.* have shown that solubilisation of the enzyme from the membranes can also change the ligand-binding properties of CYP51s^38^. Assays using *A. fumigatus* NADPH cytochrome P450 reductase reconstituted with membrane preparations of *A. fumigatus* CYP51A or CYP51B enzymes heterologously expressed in *E. coli* gave IC_50_ values 34-fold higher for FLC binding to AfCYP51A (17 μM) compared to AfCYP51B (0.5 μM). In contrast, FLC binding by these two CYP51 enzymes estimated using type II binding with the Ni-NTA affinity purified enzymes gave only a 3-fold difference in *K*_*d*_ values i.e. 12 μM for AfCYP51A and 4 μM for AfCYP51B. These discrepancies, presumably caused by the solubilisation of the enzyme with detergents, highlight the importance of the lipid bilayer in positioning the enzyme, determining its conformation and the access of triazole inhibitors to the active site. Reconstitution of the affinity-purified enzyme in lipid vesicles or nanodisks^39^ may provide methods to obtain more reliable and physiologically relevant estimates of the binding affinities of CYP51 inhibitors.

The ScErg11p6 × His Y140F/H mutations disrupt the water-mediated hydrogen bond network between enzyme and both FLC and VCZ. These findings help explain the FLC and VCZ resistance but retention of ITC and PCZ susceptibility in Y140F/H mutants. The key tertiary hydroxyl group in FLC and VCZ is replaced by a 1,3-dioxolane moiety in both long-tailed triazoles with additional hydrophobic interactions with the entry channel exclusive to these inhibitors. Calorimetric studies have shown that it is energetically favourable to include a water molecule at the protein-ligand interface, providing evidence that water-mediated interactions can stabilise protein-ligand complexes^40^. Sharrow *et al.* demonstrated that the loss of water-mediated hydrogen bonding due to a tyrosine to phenylalanine mutation reduced favourable enthalpy and the binding affinity of a ligand to its target protein^41^. The water-mediated polar interactions between Y140 and the tertiary hydroxyl of FLC and VCZ optimally stabilise the binding of these drugs.

Triazole drugs currently in clinical trials include albaconazole, ravuconazole, isavuconazole^42^ and VT-1161^16^,^43^ (Supplementary Fig. S6). All four of these compounds have the tertiary hydroxyl group that would interact with the Y140 residue. We postulate that these drugs may become less effective inhibitors of CYP51 due to mutations equivalent to Y140F/H (*S. cerevisiae* numbering). Isavuconazole has been approved for treatment of invasive aspergillosis in the USA^44^. However clinical isolates of *A. fumigatus* with the TR_46_/Y121F/T289A CYP51 mutations are found in many parts of the world^45^,^46^,^47^. This mutant has the potential to be resistant to isavuconazole due to the Y121F mutation, which confers resistance to VCZ^25^,^26^. The novel tetrazole compound VT-1161 is currently in phase II clinical trials and has been shown to be a potent inhibitor of *C. albicans* CYP51^16^. A recent structure of the *Trypanosoma cruzi* CYP51 in complex with VT-1161 (PDB ID: 5AJR)^48^ places the hydroxyl group of the drug into the same position as the hydroxyl group of FLC in the ScErg11p structure (PDB ID: 4WMZ). Thus the tertiary hydroxyl of VT-1161 could interact with Y140 (*S. cerevisiae* numbering) via a water mediated hydrogen bond network. Therefore, mutations equivalent to ScErg11p Y140F/H have the potential to confer resistance to albaconazole, ravuconazole, isavuconazole or VT-1161 unless their medium-length tails in the entry channel confer sufficient affinity to counteract this problem. The short-tailed agrochemicals tebuconazole and prothioconazole have a similarly located hydroxyl group and resistance can be expected to be conferred via the equivalent mutations in fungal phytopathogens e.g. *Z. tritici* CYP51 Y137F and *P. pachyrhizi* CYP51 Y131F I475T.

In conclusion, ScErg11p6 × His crystal structures have revealed that the resistance conferred by the Y140F/H mutations via the loss of hydroxyl group results in the disruption of a water-mediated hydrogen bond network between the tertiary hydroxyl groups of FLC and VCZ and the enzyme. This leads directly to a decrease in binding affinity. We predict that this type of resistance could be avoided by substituting the hydroxyl of group of FLC and VCZ with the 1,3-dioxolanyl group found in ITC or PCZ in order to minimise those water-mediated interactions. The design of azole drugs with tails that interact more effectively with the substrate entry channel than FLC and VCZ may also help avoid this problem.

## Methods

### Yeast strains and culture media

The *S. cerevisiae* AD2Δ host was used to create the strains used in this study. The strain was made by deletion of the *HIS1* gene from the previously described ADΔ^49^ strain. ADΔ and AD2Δ lack 7 major ATP-binding cassette transporters and the *PDR3* gene. Both strains have the gain-of function *pdr1–3* mutation in the *PDR1* transcriptional regulator gene to provide constitutive overexpression of ScErg11p6 × His from the *PDR5* locus. Yeast strains were grown on YPD medium: 1% (wt/vol) Bacto-yeast extract (BD Difco™ Laboratories Inc, Franklin Lakes, NJ), 2% (wt/vol) Bacto-peptone (BD Difco™) and 2% (wt/vol) glucose. Synthetic defined (SD) medium was used for selection of transformants. It contained 2% (wt/vol) glucose, 0.67% (wt/vol) yeast nitrogen base without amino acids (BD Difco™), 2% (wt/vol) agar (Oxoid Ltd., Hampshire, UK) and either uracil drop-out (Qbiogene, Irvine, CA) or histidine drop-out (Formedium™, Norfolk, UK) complete supplement mixture. Liquid SD media with complete supplement mixture (Formedium™) containing 10 mM MES and 20 mM HEPES buffered with TRIS to pH 6.8 was used for MIC_80_ determinations.

### Materials

Desalted oligonucleotides, FLC, ITC, VCZ and PCZ were purchased from Sigma-Aldrich Ltd (St. Louis, MO). Colony polymerase chain reactions (PCR) were carried out using TaKaRa DNA polymerase (Takara Bio Inc, Shiga, Japan). All other PCR reactions were performed using KOD Hot Start DNA polymerase (Novagen, Madison, WI). PCR clean up and DNA gel extraction was carried out using kits from Qiagen Pty Ltd (Limburg, Netherlands). Genomic DNA from yeast was isolated using the Y-DER kit from Thermo Fisher (Waltham, MA). Yeast DNA transformation was carried out using an Alkali Cation Yeast Transformation kit from Qbiogene (Irvine, CA). DNA transformation cassettes and genes inserted at the *PDR5* locus were confirmed by DNA sequence analysis performed at Genetic Analysis Services facility (University of Otago, Dunedin, New Zealand). The presence of the mutations Y140F/H in ScErg11p6 × His was verified by mass spectrometry at the Centre for Protein Research (University of Otago, New Zealand). An LTQ-Orbitrap hybrid mass spectrometer was used to obtain protein sequence coverage of at least 78% for each construct (Supplementary Figs S1 and S2).

### Construction of yeast strains overexpressing ScErg11p6 × His Y140F/H

ScErg11p Y140F/H constructs were made by recombinant PCR using genomic DNA from the ADΔ ScErg11p6 × His overexpressing strain (Supplementary Table S1) as template. Together with standard flanking primers (PDR5F GAACATGAACGTTCCTCAGCGCG and PDR5DS TATGAGAAGACGGTTCGCCATTCGGACAG) the forward primer ScErg11_Y140F_f (AAGGTGTTATT**TTC**GATTGTCCAAATTC) and the reverse primer ScErg11_Y140F_r (TTGGACAATC**GAA**AATAACACCTTTACC) were used to create fragments for recombinant PCR to introduce the Y140F mutation. Forward primer ScErg11_Y140H_f (AAGGTGTTATT**CAT**GATTGTCCAAATTC) and reverse primer ScErg11_Y140H_r (TTGGACAATC**ATG**AATAACACCTTTACC) were used to introduce the Y140H mutation. The AD2Δ strain was transformed with linear mutant *ScERG11* DNA constructs that included a C-terminal hexahistidine tag, the PGK transcription terminator, a *URA3* selection marker, bordered by sequences from the *PDR5* locus for integration into the genome via homologous recombination as described by Lamping *et al.*^49^. Transformants were selected using SD-Ura agar plates incubated for 48–72 hrs at 30 °C. Colony PCR was performed on the resultant transformants to identify clones with the insert in the correct position. The *ScERG11* open reading frame and the presence of the expected mutation were confirmed by DNA sequence analysis. The resulting strains were denoted AD2ΔScErg11_Y140F and AD2ΔScErg11_Y140H (Supplementary Table S1). The endogenous *ScERG11* was deleted from mutant strains by replacement with a disruption cassette containing the *His1* marker^32^. Transformants were selected on SD-His agar plates. Colony PCR and DNA sequence analysis were used to confirm the correct inserts. The resulting strains were designated AD3ΔScErg11_Y140F and AD3ΔScErg11_Y140H (Supplementary Table S1).

### Azole susceptibility of strains overexpressing ScErg11p

The susceptibilities of strains overexpressing wild type ScErg11p6 × His and ScErg11p6 × His Y140F/H to triazole drugs were measured as MIC_80_ values using broth microdilution assays. The MIC_80_s were defined as 80% growth inhibition compared to no drug controls because triazole drugs are fungistatic rather than fungicidal and can give trailing growth. MIC_80_s to FLC, ITC and VCZ were determined in 96-well microtiter plates using SD buffered to pH 6.8 instead of RPMI^50^. Cells were seeded at OD_600nm_ = 0.005 (1.5 × 10^4^ CFU) and the plates were incubated in 30 °C with shaking at 200 rpm for 48 hrs. Cell growth was assessed by measuring the OD_600nm_ of wells using a BioTek Synergy™ 2 multi-mode plate reader (BioTek Instruments, Vermont, USA). Each MIC_80_ was determined using triplicate measurements for three clones of each strain in three separate experiments.

### Purification of ScErg11p

The purification of Y140F/H mutant ScErg11p6 × His was carried out according to the methods described previously by Monk *et al.*^31^ using strains deleted of native *ERG11 i.e.* AD3ΔScErg11_Y140F and AD3ΔScErg11_Y140H. In brief, yeast cells were grown in 1.5 L liquid cultures in YPD medium to OD_600nm_ ~10 at 30 °C with shaking at 200 rpm. Harvested yeast cells were broken using a bead beating protocol and crude membranes were prepared by differential centrifugation. The protein concentration of crude membranes were estimated using the Lowry method^51^ with bovine serum albumin (Thermo Fisher) as standard. Crude membranes were solubilised with 10× critical micelle concentration (CMC) *n*-decyl-β-D-maltoside (DM) in a medium containing 10% (wt/vol) glycerol, 250 mM NaCl, 20 mM Tris pH 7.5, 0.5 mM phenylmethanesulfonyl fluoride (PMSF) and 1 EDTA-free protease inhibitor pill (Roche) per 200 mL. The solubilised ScErg11p6 × His was affinity-purified using 2 mL of packed Ni-NTA-agarose matrix (Qiagen) per 1 gm of crude membrane protein. Affinity purification buffer containing 10% (wt/vol) glycerol, 250 mM NaCl, 20 mM Tris pH 7.5, 0.5 mM PMSF, 16 mM (10 × CMC) DM, 20 mM imidazole and 1 EDTA-free protease inhibitor pill per 200 mL was used to wash non-specifically bound proteins from the column. The ScErg11p6 × His enzyme was eluted from the column by using 200 mM imidazole in the affinity purification buffer. The triazole drugs ITC, FLC, VCZ or PCZ dissolved in dimethyl sulfoxide (DMSO) were added to the pooled fractions with final concentrations of 40 μM for FLC and VCZ and 20 μM for PCZ and ITC.

Affinity-purified fractions were further purified by size exclusion chromatography (SEC) using a Superdex 200 10/300 GL column (GE Healthcare Life Sciences, UK). The column was equilibrated with degassed and filtered SEC buffer which contained 10% (wt/vol) glycerol, 150 mM NaCl, 20 mM HEPES, 0.5 mM PMSF, 6.4 mM DM (4 × CMC) and 2 EDTA-free protease inhibitor pills per 400 ml pH adjusted with NaOH to 7.5 at room temperature. The appropriate drug was added to the SEC buffer for co-purification; 10 μM FLC, 10 μM VCZ, 2 μM ITC or 2 μM PCZ. The coloured fractions containing the 62 kDa ScErg11p6 × His were pooled and concentrated using 50 kDa molecular-weight cut-off Amicon Ultra-4 centrifugal filters (Merck Millipore Ltd, Cork, Ireland).

### Crystallisation and data collection

A hanging-drop vapour-diffusion method was used to crystallise the ScErg11p6 × His Y140F/H co-purified with a triazole drug. The reservoir solution contained 45% polyethylene glycol-400 in 100 mM glycine at a pH range of 9.3–9.55. Drops (total volume 4 μl) were in a 1:1 ratio of reservoir solution and ~20 mg/ml of the protein in SEC buffer. Red boat-shaped crystals formed after about one week of incubation at 18 °C and were collected for X-ray studies. The crystals were flash-cooled in liquid nitrogen prior to data collection. Datasets were collected on the MX1 beamline (ADSC Quantum 210r detector) or MX2 microbeam (ADSC Quantum 315r detector) at the Australian Synchrotron (Melbourne, Australia). During data collection the crystals were kept frozen under a cryostream at −180 °C. Indexing and integration of data was done using iMosflm^52^ and scaling with SCALA^53^. Phaser-MR^54^ from Phenix was used to carry out molecular replacement using ScErg11p6 × His complexed with lanosterol (PDB ID: 4LXJ) as a template^31^. Refinement and modelling were performed using phenix.refine^55^ and Coot^56^. Waters were added if at least one hydrogen bond was detected (2.5–3.3 Å) and the inhibitors were modelled into the appropriate density in the active site and substrate channel. The crystallographic information files (.cif) for triazole inhibitors were obtained from the Grade Global Phasing online tool (Global Phasing Ltd.). During refinement Fe – nitrogen and Fe – sulfur distances were constrained to 2.15 Å and 2.33 Å based on the average coordinate bond distance of more than 80 known Fe-N (triazole) complexes and 4 heme Fe-S in the Cambridge Structural Database^57^ as previously described^32^. The Ramachandran statistics for residues range from 95.45–97.15% for residues in preferred regions, 2.66–3.98% for residues in allowed regions and 0.38–2.15% for residues in disallowed regions for all datasets.

### Spectral binding characteristics of Y140F/H mutants

The concentration of functional cytochrome P450 for drug binding studies was determined using the carbon monoxide binding spectra according to the protocol described by Guengerich *et al.*^33^. Affinity purified ScErg11p6 × His used for spectroscopic assays was eluted using affinity purification buffer containing 50 mM L-histidine instead of imidazole^37^. L-histidine was removed from the sample by washing the enzyme with solubilisation buffer containing 16 mM DM using 50 kDa molecular-weight cut-off Amicon Ultra-4 centrifugal filters (Merck Millipore Ltd, Cork, Ireland). The removal of l-histidine was checked by taking the absolute spectra of the sample using the Ultrospec™ 6300 pro UV/Visible spectrophotometer. The heme peak for wild type protein with no ligand was at ~417 nm. With L-histidine bound the peak was detected at ~420 nm. Enzyme concentration was determined by saturating the sample cuvette with CO gas prior to the addition of sodium dithionite. The reference cuvette containing the same amount of enzyme was treated with sodium dithionite only. The P450 concentration was determined by measuring the difference in absorbance between 446 and 490 nm and using the extinction coefficient of 91 mM^−1 ^cm^−1^ 58. Absorption spectra were recorded with a Cary 1 Bio UV-visible spectrophotometer using 10 mm path UV transparent plastic cuvettes (GE Healthcare Life Sciences, UK). Difference spectra were measured using 1 μM ScErg11p6 × His wild type or Y140F/H enzyme titrated with the triazole drugs ITC, PCZ, VCZ and FLC. Triazole drugs dissolved in DMSO were added to the sample cuvette, with the same amount of DMSO added to the reference cuvette. The total amount of DMSO was <2% of the total volume in the cuvette. Difference spectra between 350–500 nm were recorded and the trough-peak absorbance changes were used to plot binding curves. The dissociation constant *K*_d_ for type II binding of triazole drugs was calculated using GraphPad Prism 6 Software (GraphPad Prism, San Diego, CA) by applying the Hill equation using the formula ΔA = ΔA_max_ [Azole]^*n*^/([Azole]^*n*^ + *K*_*d*_^*n*^), with ΔA_max_ being the maximum change in absorbance and [Azole] the azole concentration.

## Additional Information

**How to cite this article**: Sagatova, A. A. *et al.* Triazole resistance mediated by mutations of a conserved active site tyrosine in fungal lanosterol 14α-demethylase. *Sci. Rep.* **6**, 26213; doi: 10.1038/srep26213 (2016).

## Supplementary Material

Supplementary Information

## Figures and Tables

**Figure 1:**
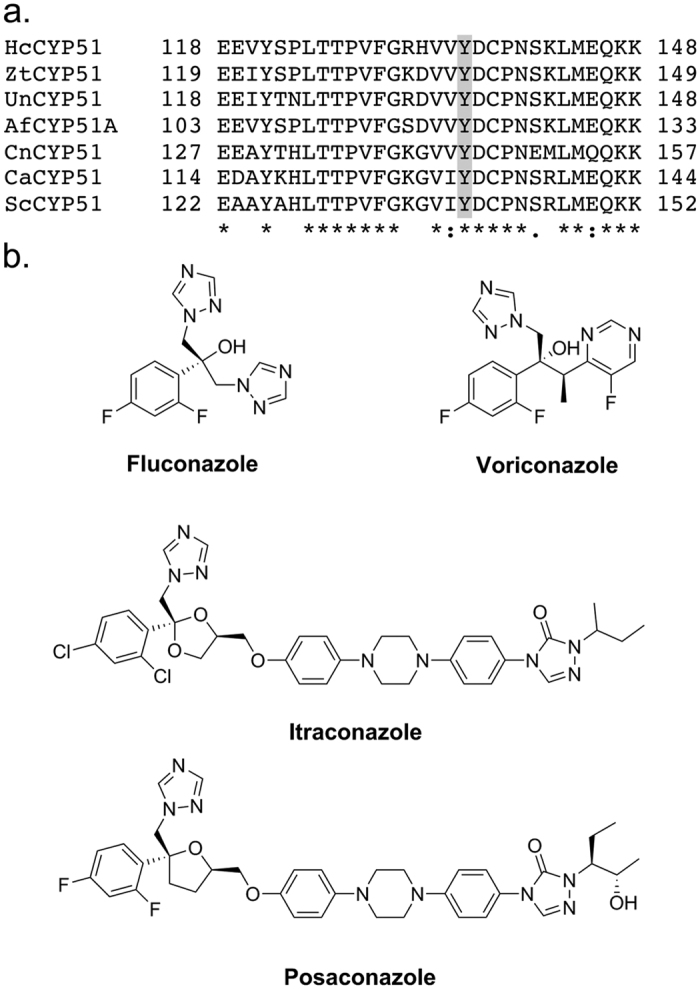
(**a**) Sequence alignment of fungal CYP51s. Alignment of *Histoplasma capsulatum* CYP51 (HcCYP51), *Zymoseptoria tritici* CYP51 (ZtCYP51), *Uncinula necator* CYP51 (UnCYP51), *Aspergillus fumigatus* CYP51A (AfCYP51A), *Cryptococcus neoformans* CYP51 (CnCYP51), *Candida albicans* CYP51 (CaErg11p), and *Saccharomyces cerevisiae* CYP51 (ScCYP51). The frequently mutated tyrosine residue homologous to ScErg11p Y140 is highlighted in grey. (**b**) The chemical structures of triazole antifungals used in this study: fluconazole, voriconazole, itraconazole and posaconazole.

**Table 1:** Triazole binding to affinity purified wild type and ScErg11p6 × His Y140F/H.

ScErg11p	Triazole	[Azole]_0.5_	*K*_d_ (μM)	Hill number
Wild-type	FLC	0.43	0.074 (±0.015)	3.0
	VCZ	0.40	0.082 (±0.018)	2.7
	ITC	0.28	0.123 (±0.027)	1.6
	PCZ	0.32	0.078 (±0.023)	2.2
Y140F	FLC	0.33	0.13 (±0.05)	1.86
	VCZ	0.27	0.03 (±0.02)	2.60
	ITC	0.33	0.13 (±0.07)	1.83
	PCZ	0.29	0.15 (±0.07)	1.57
Y140H	FLC	0.27	0.11 (±0.06)	1.68
	VCZ	0.29	0.22 (±0.07)	1.23
	ITC	0.25	0.13 (±0.06)	1.48
	PCZ	0.23	0.10 (±0.04)	1.57

Values in brackets indicate standard errors. IC_50_ value is denoted as [Azole]_0.5_.
