# Supplementary material for: Triazole resistance mediated by mutations of a conserved active site tyrosine in fungal lanosterol 14α-demethylase
Source: Sci Rep. 2016 May 18;6:26213. doi: 10.1038/srep26213 (PMC4870556; doi:10.1038/srep26213)
Supplement: Supplementary Information [file srep26213-s1.pdf]

Supplementary Information

Triazole resistance mediated by mutations of a conserved active site tyrosine in fungal lanosterol 14α-demethylase

Alia A. Sagatova, Mikhail V. Keniya, Rajni K. Wilson, Manya Sabherwal, Joel D. A. Tyndall* and Brian C. Monk*

*Address correspondence to Brian C. Monk brian.monk@otago.ac.nz or Joel D. A. Tyndall joel.tyndall@otago.ac.nz

Supplementary Table S1. Yeast strains used in this study.

Strain	Genotype	Reference
**ADΔ**	MATα *PDR1-3 Δyor1::hisG Δsnq2::hisG Δpdr3::hisG Δpdr10::hisG Δpdr11::hisG Δycf1::hisG Δpdr5::hisG Δpdr15::hisG Δura3 Δhis*AD124567 *Δpdr5*::*hisG Δpdr15*::*hisG*, *Δura3*	Lamping *et al.*[1]
**AD2Δ**	ADΔ, *Δhis1*:*dpl200*	Sagatova *et al.*[2]
**AD3ΔScErg11p**	AD2Δ, *Δpdr5*::ScERG11-URA3, *Δerg11*::HIS1	Sagatova *et al.*[2]
**AD2ΔScErg11p Y140F**	AD2Δ, *Δpdr5*::ScERG11_Y140F-URA3	This study
**AD3ΔScErg11p Y140F**	AD2Δ *Δpdr5*::ScERG11_Y140F, *Δerg11*::HIS1	This study
**AD2ΔScErg11p Y140H**	AD2Δ, *Δpdr5*::ScERG11_Y140H-URA3	This study
**AD3ΔScErg11p Y140H**	AD2Δ *Δpdr5*::ScERG11_Y140H, *Δerg11*::HIS1	This study

Supplementary Table S2. MIC_{80} values of mutant and wild type strains.

Strain	MIC_{80} (μg/ml)		
	FLC	VCZ	ITC
**AD3ΔScErg11p**	2.1 (±0.02)	0.25 (±0.050)	0.105 (±0.004)
**AD3ΔScErg11p_Y140F**	4.0 (±0.20)	0.42 (±0.016)	0.090 (±0.006)
**AD3ΔScErg11p_Y140H**	3.4 (±0.02)	0.29 (±0.002)	0.077 (±0.006)

The table shows the mean for 3 separate clones of each strain using data obtained in triplicate measurements from at least 3 different experiments (a total of 9 determinations per strain). SEM is indicated in brackets.

Supplementary Table S3. Characteristics of Type II difference spectra of ScErg11p6×His wild type and Y140F/H mutants

ScErg11p	Wild-type			Y140F			Y140H		
**Triazole**	ΔA_{max}	λ_{trough}	λ_{peak}	ΔA_{max}	λ_{trough}	λ_{peak}	ΔA_{max}	λ_{trough}	λ_{peak}
**FLC**	0.048	410	428	0.023	406	425	0.016	410	428
**VCZ**	0.051	410	428	0.019	408	424	0.018	410	424
**ITC**	0.037	410	428	0.014	406	425	0.016	406	424
**PCZ**	0.031	410	428	0.016	410	424	0.016	409	424

Ni-NTA affinity purified enzyme: Wild type = AD3ΔScErg11p; Y140F = AD3ΔScErg11p Y140F; Y140H = AD3ΔScErg11p Y140H.

Supplementary Table S4. Data collection and refinement statistics for ScErg11p6×His Y140F mutant.

PDB ID	4ZDY	4ZE1	4ZDZ	4ZE0
**Ligand**	ITC	PCZ	FLC	VCZ
**Data collection**				
Wavelength (Å)	0.9537	0.9537	0.9537	0.9537
Space group	$P\ 1\ 2_1\ 1$	$P\ 1\ 2_1\ 1$	$P\ 1\ 2_1\ 1$	$P\ 1\ 2_1\ 1$
Cell dimensions				
a, *b*, *c* (Å)	78.18, 67.07, 80.70	78.79, 67.75, 81.06	78.91, 67.15, 81.14	77.22, 66.55, 80.90
α, β, γ (°)	90, 98.93, 90	90, 99.43, 90	90, 99.39, 90	90, 98.67, 90
Total reflections	384013	285878	125003	251724
Unique reflections	54071	52170	37123	40138
Resolution (Å)	34.27 – 2.02	37.45 – 2.05	51.75 – 2.30	51.48 – 2.20
	(2.13 – 2.02)*	(2.11 – 2.05)	(2.38 – 2.30)	(2.27 – 2.20)
R_{merge}	0.087 (0.648)	0.082 (0.979)	0.151 (0.456)	0.113 (0.853)
$I / \sigma I$	15.9 (3.3)	12.9 (1.8)	3.9 (1.5)	10.8 (2.0)
Completeness (%)	99.6 (98.2)	98.3 (98.0)	99.1 (99.5)	97.4 (92.7)
Redundancy	7.1 (6.8)	5.5 (5.5)	3.4 (3.3)	6.2 (5.5)
$CC_{1/2}$	0.999 (0.858)	0.997 (0.538)	0.970 (0.860)	0.995 (0.907)
**Refinement**				
Resolution (Å)	2.02	2.05	2.30	2.20
No. reflections	54031	52115	37100	40220
$R_{work} / R_{free}^{\S}$	0.191 / 0.229	0.202 / 0.243	0.197 / 0.237	0.192 / 0.239
No. atoms				
Protein	4312	4323	4319	4337
Ligand/ion	49	51	22	25
Water	187	139	191	172
B-factors ($Å^2$)				
Protein	35.3	50.0	41.4	41.9
Ligand/ion	27.9	43.0	26.9	27.6
Water	34.1	45.1	39.2	39.9
R.m.s. deviations				
Bond lengths (Å)	0.008	0.008	0.005	0.008
Bond angles (°)	1.0	1.1	0.9	1.1

* Values in parentheses are for highest-resolution shell. One crystal was used per structure. § R_{free} was computed using a test set composed of 5% of data.

Supplementary Table S5. Data collection and refinement statistics for ScErg11p6×His wild type and Y140H mutant.

PDB ID	4ZE2	4ZE3	5HS1[a]
**Ligand**	ITC	FLC	VCZ
**Data collection**			
Wavelength (Å)	0.9537	0.9537	0.9537
Space group	P 1 2_1 1	P 1 2_1 1	P 1 2_1 1
Cell dimensions			
a, *b*, *c* (Å)	77.83, 66.67, 81.01	76.49, 64.84, 81.03	78.01, 67.32, 81.09
α, β, γ (°)	90, 98.88, 90	90, 98.18, 90	90, 98.67, 90
Total reflections	144675	153351	94492
Unique reflections	36597	39850	48408
Resolution (Å)	80.04 – 2.30 (2.38 – 2.30)*	80.21 – 2.20 (2.27 – 2.20)	51.57 - 2.10 (2.16 - 2.10)
R_{merge}	0.087 (0.643)	0.064 (0.628)	0.084 (0.590)
$I / \sigma I$	8.7 (1.9)	11.4 (2.2)	7.0 (1.5)
Completeness (%)	99.8 (100.0)	99.3 (98.3)	99.5 (98.4)
Redundancy	4.0 (4.0)	3.8 (3.8)	3.7 (3.4)
$CC_{1/2}$	0.994 (0.752)	0.997 (0.718)	0.998 (0.768)
**Refinement**			
Resolution (Å)	2.3	2.2	2.1
No. reflections	36597	39809	48423
R_{work} / R_{free}[§]	0.188 / 0.232	0.192 / 0.227	0.204 / 0.238
No. atoms			
Protein	4325	4436	4271
Ligand/ion	49	22	68
Water	110	120	167
B-factors (Å^2)			
Protein	49.0	44.3	42.4
Ligand/ion	40.9	28.4	27.6
Water	44.3	42.2	39.7
R.m.s. deviations			
Bond lengths (Å)	0.009	0.004	0.008
Bond angles (°)	1.2	0.9	0.9

Values in parentheses are for highest-resolution shell. One crystal was used per structure. [a]Wild type structure. [§] R_{free} was computed using a test set composed of 5% of data.

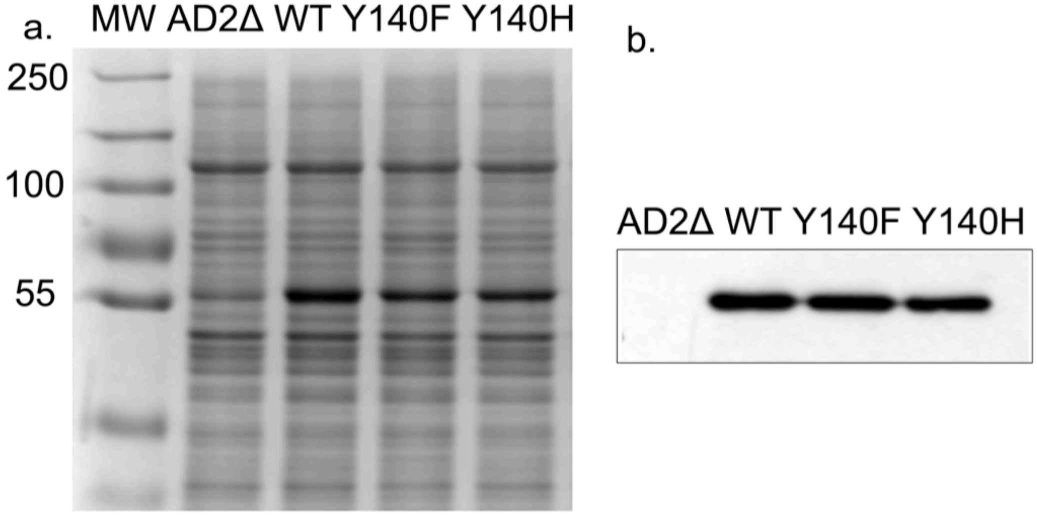

Supplementary Figure S1. SDS-PAGE and western blot analysis of ScErg11p6×His wild type (WT) and Y140F/H mutant proteins. (a) Coomassie blue R250 stained 8% acrylamide SDS-PAGE gel of samples of crude membranes (10 µg) from strains overexpressing wild type and mutant ScErg11p6×His, with the AD2Δ strain used as a negative control. Ten µL of each sample at 1 µg/µL mixed with 2 µL of 6× SDS loading dye was separated on each lane. (b) Proteins electrotransferred to nitrocellulose membranes were decorated with a mouse anti-6×His-peroxidase monoclonal antibody and visualised using chemiluminescence.

```
1   MSATKSIVGEALEYVNIGLSHFLALPLAQRISLIIIIPFI
41  YNIVWQLLYSLRKDRPPLVFYWIPWVGSAVVYGMKPYEFF
81  EECQKKYGDIFSFVLLGRVMTVYLGPKGHEFVFNAKLADV
121 SAEAAYAHLTTPVFGKGVIFDCPNSRLMEQKKFVKGALTK
161 EAFKSYVPLIAEEVYKYFRDSKNFRLNERTTGTIDVMVTQ
201 PEMTIFTASRSLLGKEMRAKLDTDFAYLYSDLDKGFTPIN
241 FVFPNLPLEHYRKRDHAQKAISGTYMSLIKERRKNNDIQD
281 RDLIDSLMKNSTYKDGVKMTDQEIANLLIGVLMGGQHTSA
321 ATSAWILLHLAERPDVQQELYEEQMRVLDGGKKELTYDLL
361 QEMPLLNQTIKETLRMHHPLHSLFRKVMKDMHVPNTSYVI
401 PAGYHVLVSPGYTHLRDEYFPNAHQFNIHRWNNDSASSYS
441 VGEEVDYGFGAISKGVSSPYLPFGGGRHRCIGEHFAYCQL
481 GVLMSIFIRTLKWHYPEGKTVPPPDFTSMVTLPTGPAKII
521 WEKRNPEQKIGGRHHHHHH
```

Supplementary Figure S2. Mass spectrometry of tryptic fingerprints for the ScErg11p6×His Y140F mutant. Protein coverage is 78%. Highlighted in grey are the peptides identified by mass spectrometry. Highlighted in red is the site of the Y to F mutation.

```
1   MSATKSIVGEALEYVNIGLSHFLALPLAQRISLIIIIPFI
41  YNIVWQLLYSLRKDRPPLVFYWIPWVGSAVVYGMKPYEFF
81  EECQKKYGDIFSFVLLGRVMTVYLGPKGHEFVFNAKLADV
121 SAEAAYAHLTTPVFGKGVIHDCPNSRLMEQKKFVKGALTK
161 EAFKSYVPLIAEEVYKYFRDSKNFRLNERTTGTIDVMVTQ
201 PEMTIFTASRSLLGKEMRAKLDTDFAYLYSDLDKGFTPIN
241 FVFPNLPLEHYRKRDHAQKAISGTYMSLIKERRKNNDIQD
281 RDLIDSLMKNSTYKDGVKMTDQEIANLLIGVLMGGQHTSA
321 ATSAWILLHLAERPDVQQELYEEQMRVLDGGKKELTYDLL
361 QEMPLLNQTIKETLRMHHPLHSLFRKVMKDMHVPNTSYVI
401 PAGYHVLVSPGYTHLRDEYFPNAHQFNIHRWNNDSASSYS
441 VGEEVDYGFGAISKGVSSPYLPFGGGRHRCIGEHFAYCQL
481 GVLMSIFIRTLKWHYPEGKTVPPPDFTSMVTLPTGPAKII
521 WEKRNPEQKIGGRHHHHHH
```

Supplementary Figure S3. Mass spectrometry of tryptic fingerprints for the ScErg11p×His Y140H mutant. Protein coverage is 93%. Highlighted in grey are the peptides identified by mass spectrometry. Highlighted in red is the site of the Y to H mutation.

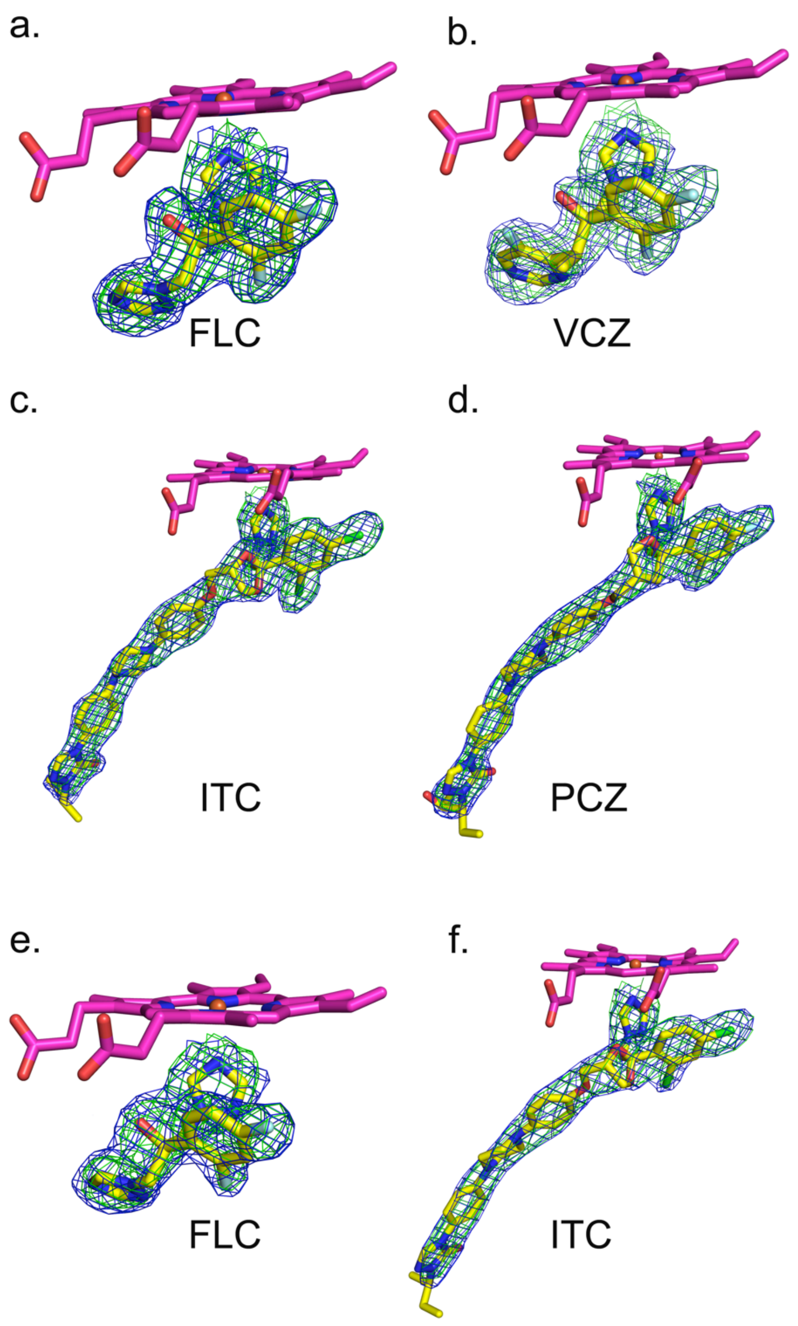

Supplementary Figure S4. OMIT maps for triazole binding to ScErg11p6×His Y140F/H mutants. Triazole drugs are shown as sticks, C atoms yellow, N atoms blue, O atoms red, Cl atoms green and F atoms pale blue. The heme is shown with C atoms in magenta. The OMIT maps $2F_o$ - F_c map (blue) are contoured at 1σ and the F_o - F_c map (green) are contoured at 3σ. Both maps were calculated using F_{calc} refined from coordinates with no ligand at the active site. The electron density is shown for FLC (2.3 Å, a), VCZ (2.2 Å, b), ITC (2.02 Å, c) and PCZ (2.05 Å, d) binding in the active site of ScErg11p6×His Y140F mutant. The FLC (2.2 Å, e) and ITC (2.3 Å, f) electron density is in the active site of ScErg116×His Y140H mutant.

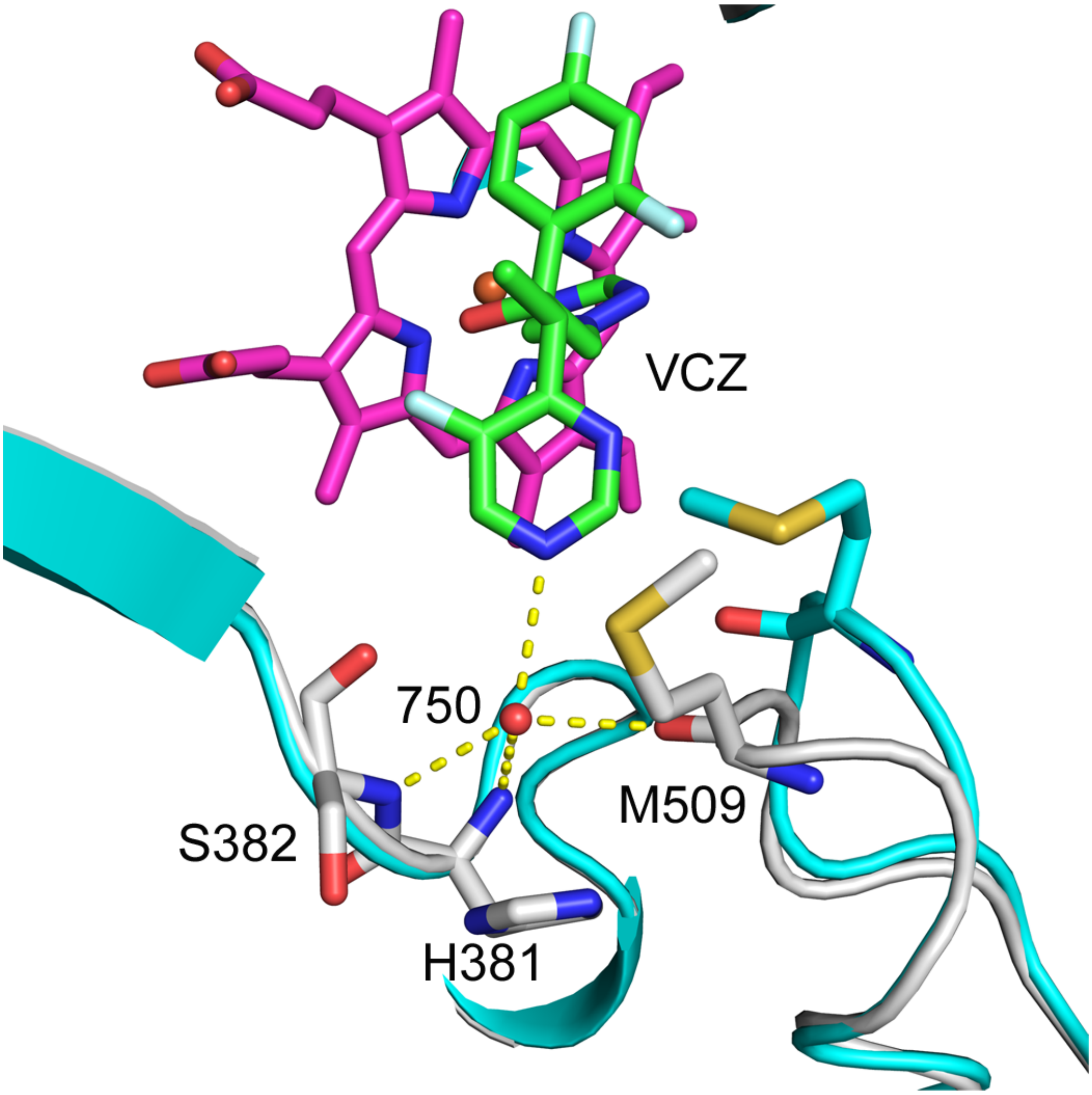

Supplementary Figure S5. Hydrogen bonding of water 750 in ScErg11p6×His complexed with VCZ and the two different positions of residue M509. The overlay of VCZ bound (PDB ID: 5HS1; grey cartoon and sticks) and the FLC bound (PDB ID: 4WMZ; cyan cartoon and sticks) ScErg11p6×His structures. VCZ but not FLC is shown. The hydrogen bonds between the pyrimidine nitrogen of VCZ and the main chain nitrogens of H381 and S382 and the carbonyl of M509 via a water molecule 750 are depicted. Hydrogen bonds are shown as yellow dashed lines and a water molecule as a red sphere. VCZ (green carbons), residues H381, S382 and M509 and the heme (magenta) are shown as sticks.

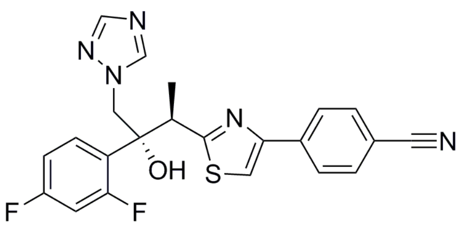

Ravuconazole

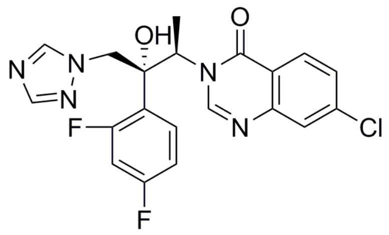

Albaconazole

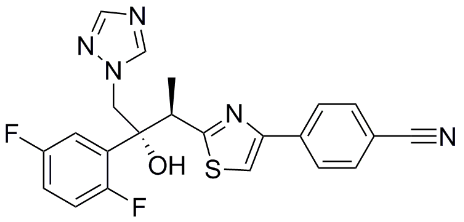

Isavuconazole

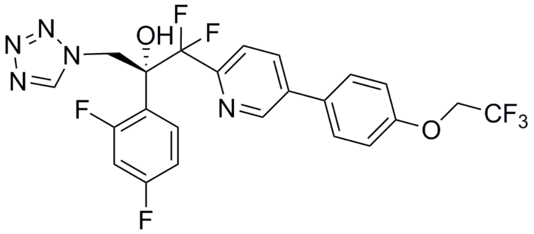

VT-1161

Supplementary Figure S6. The chemical structures of new azole drugs ravuconazole, albaconazole, isavuconazole and VT-1161.

References:

1. Lamping, E. *et al.* Characterization of three classes of membrane proteins involved in fungal azole resistance by functional hyperexpression in *Saccharomyces cerevisiae*. *Eukaryot. Cell* **6**, 1150-1165 (2007).
2. Sagatova, A., Keniya, M.V., Wilson, R.K., Monk, B.C. & Tyndall, J.D. Structural insights into binding of the antifungal drug fluconazole to *Saccharomyces cerevisiae* lanosterol 14alpha-demethylase. *Antimicrob. Agents Chemother.* (2015).
